# Bipolar Catheter Ablation as a Key for Successful Treatment of Intramural VentricuLar Arrhythmias: Results From the Bipolar‐Kiel Study

**DOI:** 10.1111/jce.70018

**Published:** 2025-09-14

**Authors:** Fabian Moser, Vera Maslova, Adrian Zaman, Thomas Demming, Martina Spehlmann, Mohammed Saad, Derk Frank, Evgeny Lian

**Affiliations:** ^1^ Department of Cardiology and Internal Intensive Medicine University Hospital Schleswig Holstein Kiel Germany

**Keywords:** bipolar ablation, premature ventricular contractions, radiofrequency ablation, ventricular arrhythmias, ventricular tachycardia

## Abstract

**Background:**

Unipolar catheter ablation (CA) is effective in the treatment of ventricular arrhythmias but its efficacy can be limited in eliminating intramural arrhythmic origins. Bipolar ablation has emerged as a bail‐out strategy. However, currently, it is being primarily utilized after a failed ablation attempt.

**Objective:**

The aim of this study is to assess the safety and efficacy of endocardial and/or epicardial bipolar catheter ablation in patients with ventricular arrhythmias within their first procedure or after a previously failed ablation attempt.

**Methods and Results:**

Patients who underwent bipolar CA between September 2022 and February 2025 were included. A total of 21 bipolar ablation procedures were performed in 19 patients (median age 66 ± 11, 11% female, median ejection fraction 42% ± 16%). In total, 16 procedures were performed due to ventricular tachycardia (VT) or VT storm, five procedures were performed due to symptomatic premature ventricular contractions (PVC). In 48% of all cases, ablation was performed within the patient's first procedure.

The interventricular septum was the most common site of bipolar ablation (11/21), followed by epi‐endocardial bipolar ablation procedures (9/21) and the papillary muscle (1/21). Bipolar ablation was successful in 17/21 patients. In one patient, AV block occurred after bipolar ablation. During a median follow‐up time of 12 ± 6 months, four patients with initially acute procedural success experienced recurrence of a ventricular arrhythmia.

**Conclusion:**

Bipolar catheter ablation is an effective tool for arrhythmias with an intramural origin. It is shown to be safe with a high acute success rate in patients undergoing their first or redo procedure for VT or PVC.

**Abbreviations**
AC: active catheter
ECG: electrocardiogram
PVC: premature ventricular contractions
RC: return catheter
RF: radiofrequency
VA: ventricular arrhythmia
VT: ventricular tachycardia

## 1 Introduction

Radiofrequency (RF) catheter ablation is a well‐established treatment option for both idiopathic and scar‐related ventricular arrhythmias (VAs). However, the efficacy of conventional RF ablation may be limited when the arrhythmogenic substrate is intramural. In such cases, unipolar RF energy delivery may not be sufficient to completely eliminate the arrhythmogenic source of the VA [1, 2, 3, 4].

Recently, bipolar ablation was presented as a technique to overcome the limitations of conventional unipolar ablation effectively reaching intramural substrate. While conventional unipolar RF catheter ablation delivers current between the catheter tip and a dispersive skin electrode, bipolar ablation delivers current between two catheters placed on either side of the target tissue. The increased current density leads thereby to improved rates of transmural lesion formation.

Several case reports and clinical series have demonstrated the use of bipolar ablation in treating VAs that have been refractory to standard unipolar ablation [5, 6, 7, 8, 9, 10, 11, 12].

However, bipolar ablation was previously reported as a bail‐out strategy, mainly after previously failed procedures. Larger retrospective studies combined small numbers of patients of different centers with different ablation approaches, using oftentimes a custom‐made equipment, with concerns about the safety of this approach [12, 13]. This led to the most recent expert consensus statement and ESC guideline recommendation of bipolar ablation being a nonstandard and investigational adjunctive ablation technique, currently only as a bailout treatment [4, 14].

The aim of this study is to assess the safety, feasibility, and efficacy of bipolar ablation in consecutive patients with VA undergoing endocardial and/or epicardial bipolar ablation within their first procedure or after a previously failed ablation attempt.

## 2 Methods

### 2.1 Study Population

Consecutive patients with ventricular tachycardia (VT) or premature ventricular contractions (PVC), undergoing bipolar RF‐based catheter ablation with deep intramural VA origin as a first or redo ablation procedure at our institution between September 2022 and February 2025 were included. Exclusion criteria were age < 18 years and patients with insufficient procedural data. Clinical characteristics and procedural outcomes of these patients were assessed. The procedures were performed by the institutional standards for RF ablation. All patients provided written informed consent prior to the ablation procedure and were included in the Catheter Ablation registry. The registry was approved by the Local Ethics Committee and is a prospective, observational clinical cohort that enrolls patients with heart rhythm disorders undergoing catheter ablation (AZ: D418/23).

### 2.2 Procedural Management

Transthoracic echocardiography was performed before the procedure to assess left ventricular (LV) function, valve disease, septal wall thickness, and to exclude ventricular thrombi. Additional preprocedural imaging using magnetic resonance imaging or computed tomography was performed when required by preceding diagnostics. Antiarrhythmics were discontinued for five half‐lives before the procedure whenever possible. In patients treated with novel oral anticoagulants, anticoagulation was interrupted for 12 h before the procedure. The procedure was performed under conscious sedation using propofol and fentanyl.

After vascular access, a multipolar catheter was positioned in the coronary sinus. In patients undergoing CA for VT, an additional multipolar catheter was positioned in the right ventricular apex.

Mapping was guided by an electroanatomic mapping system (CARTO Biosense Webster, Diamond Bar, CA, USA; Ensite Abbott, St Paul, MN, USA; Rhythmia Boston Scientific, Natick, MA, USA) Mapping was performed with a multielectrode mapping catheter (Advisor HD Grid Catheter, Abbott; PENTARAY, Biosense Webster or the 64‐pole Orion catheter Boston Scientific, Marlborough, MA, USA) in patients undergoing VT ablation or a single tip mapping an ablation catheter (TactiFlex, Abbott; THERMOCOOL SMARTTOUCH SF or QDot; Biosense Webster) in patients undergoing PVC ablation. For LV mapping, a transseptal approach using a steerable sheath (Agilis, Abbott) and/or a retrograde aortic approach was used. Heparin was administered to maintain an activated clotting time of ≥_300 s. An epicardial access using a “Tuohy” needle under fluoroscopic guidance was performed for mapping and ablation within the pericardial space.

For PVC ablation, activation mapping and/or pace mapping was used to detect the PVC origin. For VT ablation, VT induction and activation mapping was performed whenever possible and hemodynamically tolerated. For hemodynamically unstable VTs, substrate modification was performed, targeting sites identified by pace mapping, abnormal electrograms, and sites of isochronal crowding on isochronal late activation mapping. Additional coronary angiography was performed in selected cases if the suspected critical VA site was in close proximity to the coronary vessels. A distance of 10 mm or more from the ablation catheter to the major coronary arteries was considered safe for ablation, according to previous literature on unipolar ablation.

### 2.3 Intramural Ventricular Arrhythmias

The criteria for intramural VAs were defined by the following:

(1) In patients undergoing PVC ablation, mapping on opposite sides of the suspected origin showed similar activation time without detected unipolar QS‐morphology on either side and a broad area of earliest activation. (2) In patients undergoing VT ablation, the complete cycle length of the tachycardia could not be recorded despite high‐density mapping from both sides of the target area, missing diastolic potentials on either side. In a subset of patients, preprocedural imaging was performed with evidence of intramural substrates.

### 2.4 Bipolar Ablation

For bipolar ablation, two open‐irrigated ablation catheters were used as an active catheter (AC) and a return catheter (RC) with the catheters being placed at the opposite site of the target myocardium. The AC is connected to the active port of the RF generator. The RC is connected to the indifferent port of the primary generator instead of a dispersive patch using a dedicated Dr Futyma Bipolar Ablation Adapter (CorSystem, Rzeszow, Poland).

The adapter enables the connection of the RC to the indifferent port of primary generator but also to a secondary RF generator (Stockert Cordis EP Shuttle) and an individual irrigation pump. This allows temperature monitoring of both catheters individually.

The irrigation of both catheters was performed with normal saline (0.9%) with and irrigation rate of the AC according to the manufacturer's recommendations. Bipolar RF ablation was performed with a power of 20–40 W and a maximum temperature of 44°C. Contact force and contact vector of the AC was assessed throughout RF ablation to ensure adequate tissue contact.

For the AC, an open‐irrigated, contact force sensing catheter (TactiFlex, Abbott; THERMOCOOL SMARTTOUCH SF or QDot; Biosense Webster; Intellanav Stablepoint, Boston Scientific), connected to the electroanatomic mapping system, was used. For the RC, connected to the secondary RF generator, an open‐irrigated, noncontact force sensing catheter (Thermocool, Biosense Webster, Diamond Bar, CA, USA) was used. Contact force could not be measured with the RC. Baseline bipolar impedance and impedance drop were monitored.

### 2.5 Acute Outcome and Follow‐Up

Acute procedural success was defined as the absence of the clinical PVC after a waiting time of 30 min and subsequent isoproterenol/orciprenaline provocation testing during PVC ablation or noninducibility of clinical VT for VT ablation. Following ablation, all patients underwent transthoracic echocardiography at the end of the procedure, before discharge, as well as in 3‐month intervals after catheter ablation. Valve and LV systolic function, presence of pericardial effusion and the presence of a potential VSD was assessed.

Antiarrhythmic drugs were stopped after the procedure. All patients underwent prospective follow‐up within the registry. Follow‐up data were obtained from Holter ECGs, implanted devices, review of medical records, and patient consultation. Patients with an implantable cardioverter defibrillator underwent regular device interrogation at the outpatient clinic. Holter ECGs were performed at regular intervals in all patients who underwent PVC ablation. Both the patient and the referring physician were consulted regularly to assess arrhythmia recurrence and potential complications. All available information was combined to assess recurrence of the arrhythmia and to identify complications after catheter ablation.

### 2.6 Statistical Analysis

Continuous variables are presented by mean and standard deviations or median and interquartile range as appropriate. Categorical variables are presented by absolute and relative frequencies. If applicable, minimum and maximum values are also indicated.

## 3 Results

### 3.1 Patient Baseline Characteristics

A total of 21 bipolar ablation procedures in 19 patients (17 males, age 66 ± 11 years, body mass index 28 ± 5) between September 2022 and February 2025 were analyzed. Indication for catheter ablation was monomorphic PVC (*n* = 5), VT (*n* = 9), or electrical storm (*n* = 7). Underlying structural heart diseases included ischemic cardiomyopathy (*n* = 7), nonischemic cardiomyopathy (*n* = 9), and hypertrophic cardiomyopathy (*n* = 1). Ten patients (53%) presented for initial ablation, in nine patients a median of 1 previous procedure (range 1–5) of different approaches had been performed before the bipolar ablation procedure, including one patient, who previously underwent four times catheter ablation and once stereotactic radioablation. Clinical and demographic characteristics of the study population are presented in Table 1.

**Table 1:** Baseline characteristics.

Parameter	Study population
Age (years)	66 ± 11
Female sex, *n* (%)	2 (11)
Body mass index (kg/m^2^)	28.7 ± 5
LVEF, % (min; max)	42 ± 16 (10; 60)
IVS (mm)	13 ± 4
ICD present, *n* (%)	9 (43)
Antiarrhythmic therapy	
Ajmalin	1 (5)
Flecainid	0 (0)
Propafenone	0 (0)
Amiodarone	4 (21)
Sotalol	0 (0)
Mexiletine	0 (0)
Quinidine	0 (0)
Underlying condition, *n* (%)	
Ischemic cardiomyopathy	7/21 (33)
Nonischemic cardiomyopathy	9/21 (43)
Hypertrophic cardiomyopathy	1/21 (5)
Idiopathic cardiomyopathy	4/21 (19)
Procedure indication, *n* (%)	
PVC	5/21 (24)
VT	9/21 (43)
VT storm	7/21 (33)
Previous ablation, *n* (%)	10/19 (53%)
No. of previous ablation (mean, min; max)	1 (0; 5)

*Note:* Variables are expressed as absolute values, mean ± standard deviation, or median with interquartile ranges.

Abbreviations: ICD, implantable cardioverter‐defibrillator; IVS, interventricular septum; LVEF, left ventricular ejection fraction; PVC, premature ventricular contraction; SHD, structural heart disease; VA, ventricular arrhythmia; VT, ventricular tachycardia.

### 3.2 Procedural Data

Overall procedure duration was 198 ± 68 min, with a mean of 138 ± 41 min for PVC procedures and 208 ± 63 min for VT procedures. Median fluoroscopy time was 17 ± 10 min.

In one procedure, prophylactic mechanical circulatory support via Impella (Abiomed Europe, Aachen, Germany) was established before the procedure. In 12 cases (67%), access to the LV was obtained via transseptal approach, while retrograde aortic approach was performed in 4 cases (22%). In two patients (10%), both approaches were used. Subxiphoid puncture with epicardial access and three‐dimensional mapping of the epicardial surface was performed in four procedures.

Coronary angiography was performed before energy application in four procedures. Ablation points were chosen based on the earliest activation site and/or best pace map in PVC patients or suspected critical isthmus sites in VT patients.

In 18 procedures, a combined unipolar and bipolar ablation approach was used, while only bipolar ablation was carried out in the 3 cases. Unipolar ablation was performed with a maximum energy range between 20 and 50 W and with an average of 41 ± 8 W.

Procedural data are listed in Table 2.

**Table 2:** Procedural characteristics.

Parameter	Procedures
Electroanatomic mapping system, *n* (%)	
Rhythmia	3 (14)
Carto	10 (48)
Ensite	8 (38)
Procedure duration (min)	198 ± 68
Fluoroscopy time (min)	17 ± 10
Fluoroscopy dose (cGy cm^2^)	278.1 ± 346.7
Unipolar ablation in same procedure, *n* (%)	19 (91)
Mean maximum applied energy during B‐RFA (W)	30
Mean B‐RFA applications	11 ± 6
Mean B‐RFA duration	293 ± 206
VT/VT storm	16 (76)
Clinical VT inducible, *n* (%)	16 (76)
Hemodynamic support, *n* (%)	1 (5.5)
Mean B‐RFA applications	11 ± 7
Mean B‐RFA application duration	308 ± 226
PVC	5 (24)
Spontaneous PVC	5 (24)
Mean B‐RFA applications	7 ± 3
Mean B‐RFA duration	262 ± 155
Acute success	
PVC, suppression, *n* (%)	3/5 (60)
VT/VT storm, noninducibility, *n* (%)	14/16 (89)

### 3.3 Bipolar Ablation

**Figure 1:**
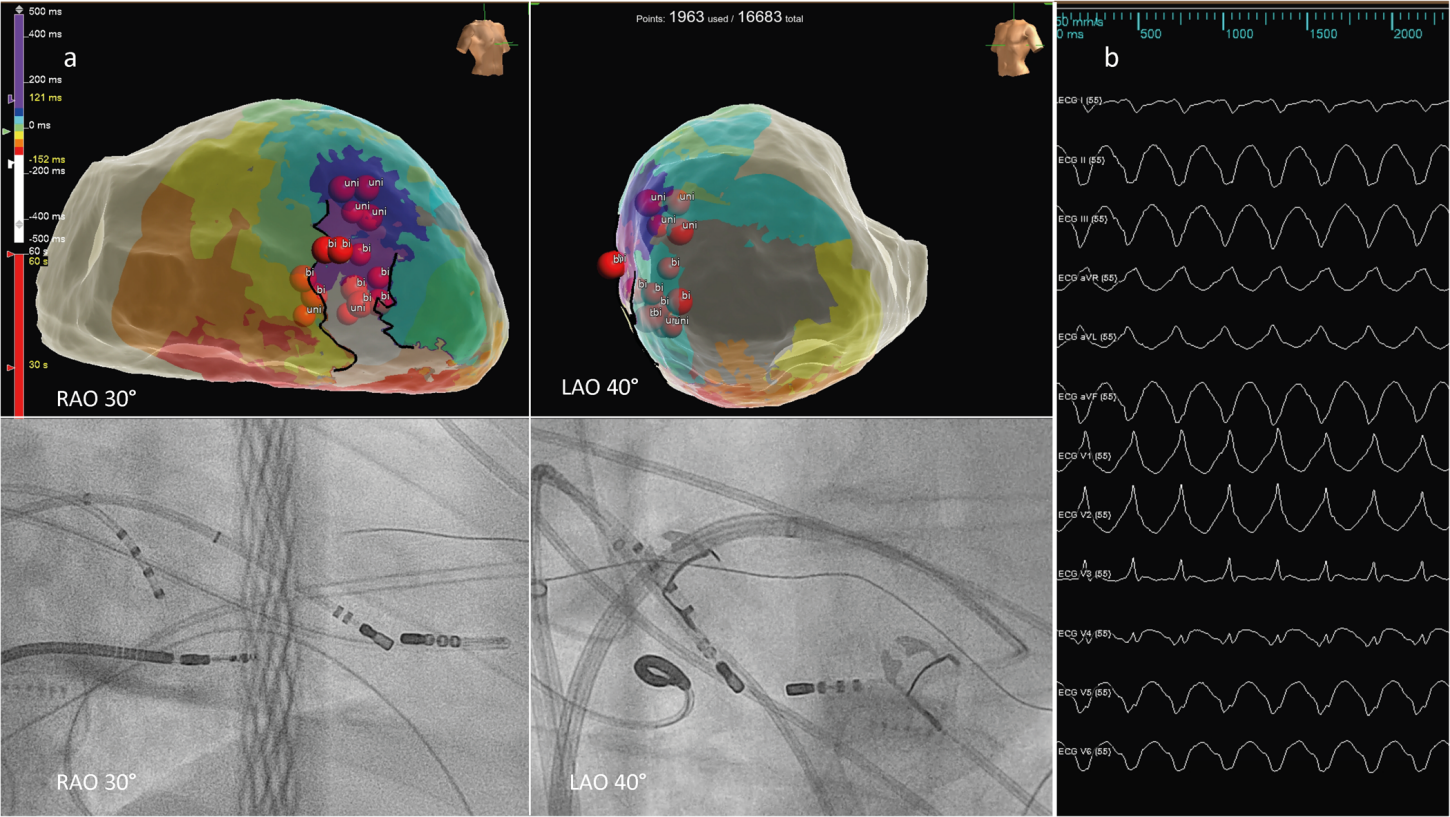
Bipolar catheter ablation of the interventricular septum. (a) Left ventricular endocardial map with uni‐ and bipolar ablation tags within the VT isthmus. (b) 12‐lead morphology of hemodynamically stable VT originating from within the interventricular septum.

**Figure 2:**
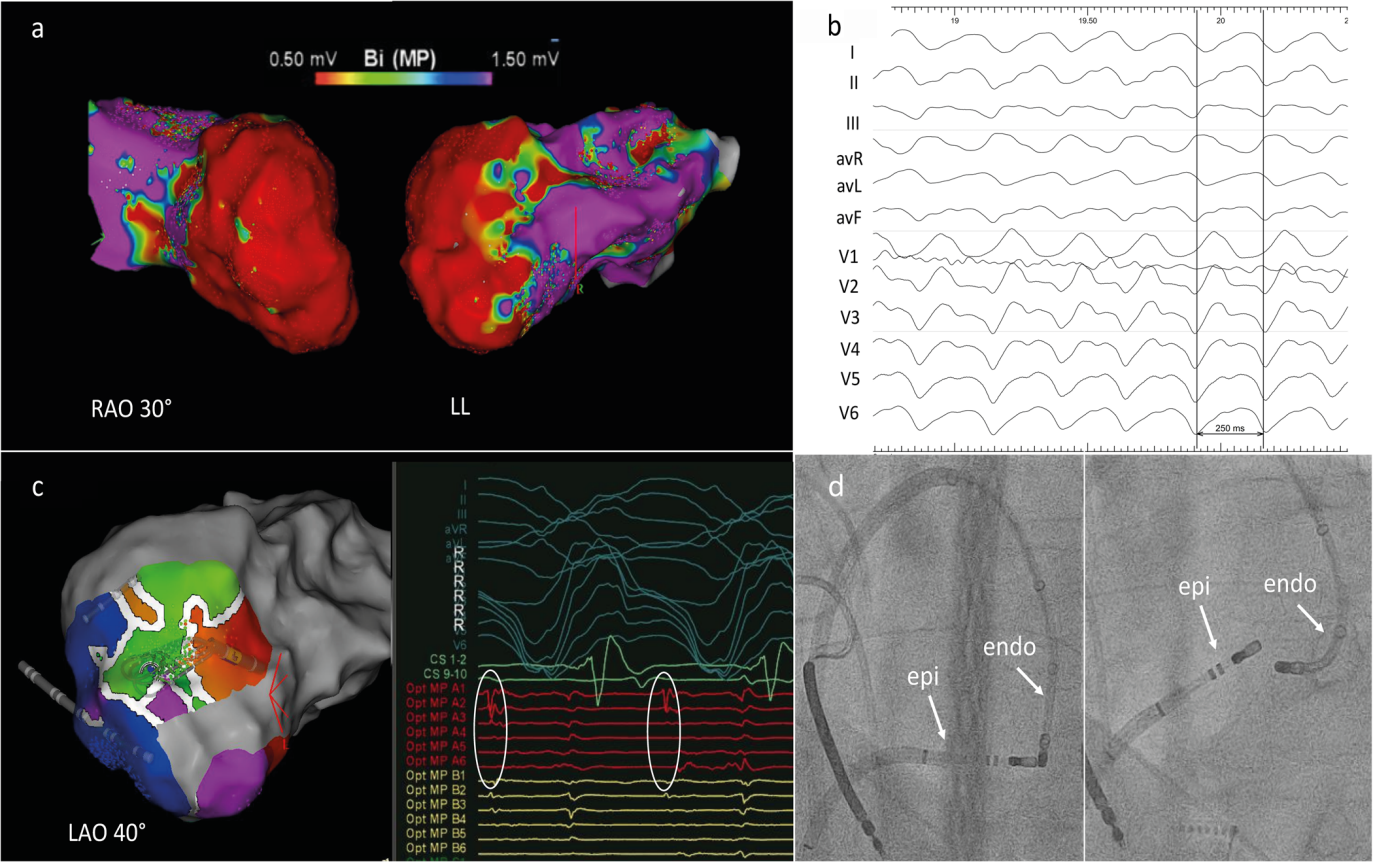
Epi‐endocardial bipolar ablation of sustained ventricular tachycardia. (a) Left ventricular endocardial voltage map in a patient with ischemic cardiomyopathy presenting with VT storm. (b) 12‐lead morphology of hemodynamically unstable VT (cycle length 250 ms). (c) High‐density mapping during VT with middiastolic potentials (Opt MP A1–A6). (d) Catheter position for the bipolar endo‐epicardial ablation.

The interventricular septum was the primary ablation target in 11 (52%) patients, with 9 of these cases originating from the posteroseptal region and 2 from the midseptal region (Figure 1 and Video 1). In five patients (31%), bipolar ablation of VA originating from the LV summit was performed. For the LV summit region, endo‐epicardial bipolar ablation between distal cardiac vein and the anterobasal LV was performed. Epicardial access ablation with subxiphoid puncture was performed in four patients (19%), with the AC being placed on the epicardial site and the RC positioned in the opposing endocardial site (Figure 2).

**Figure 3:**
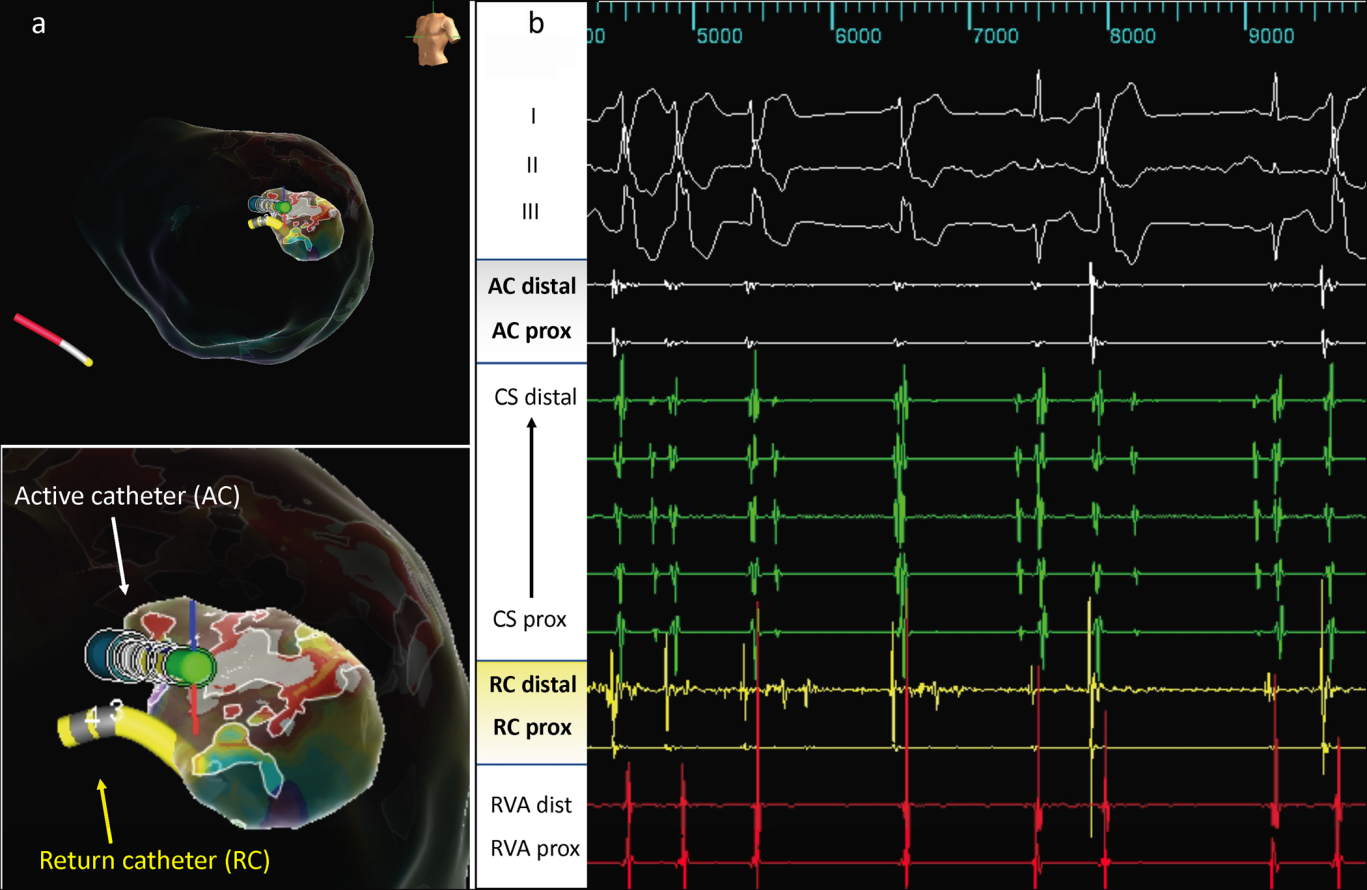
Bipolar ablation of ventricular tachycardia from anterolateral papillary muscle. (a) Left ventricular shell with endocardial activation map of VT originating from the anterolateral papillary muscle. (b) Surface‐ECG and intracardiac electrograms at time of VT termination.

In one patient (6%), a VT was targeted at the anterolateral papillary muscle with a combined transseptal and retrograde access to the LV (Figure 3).

**Figure 4:**
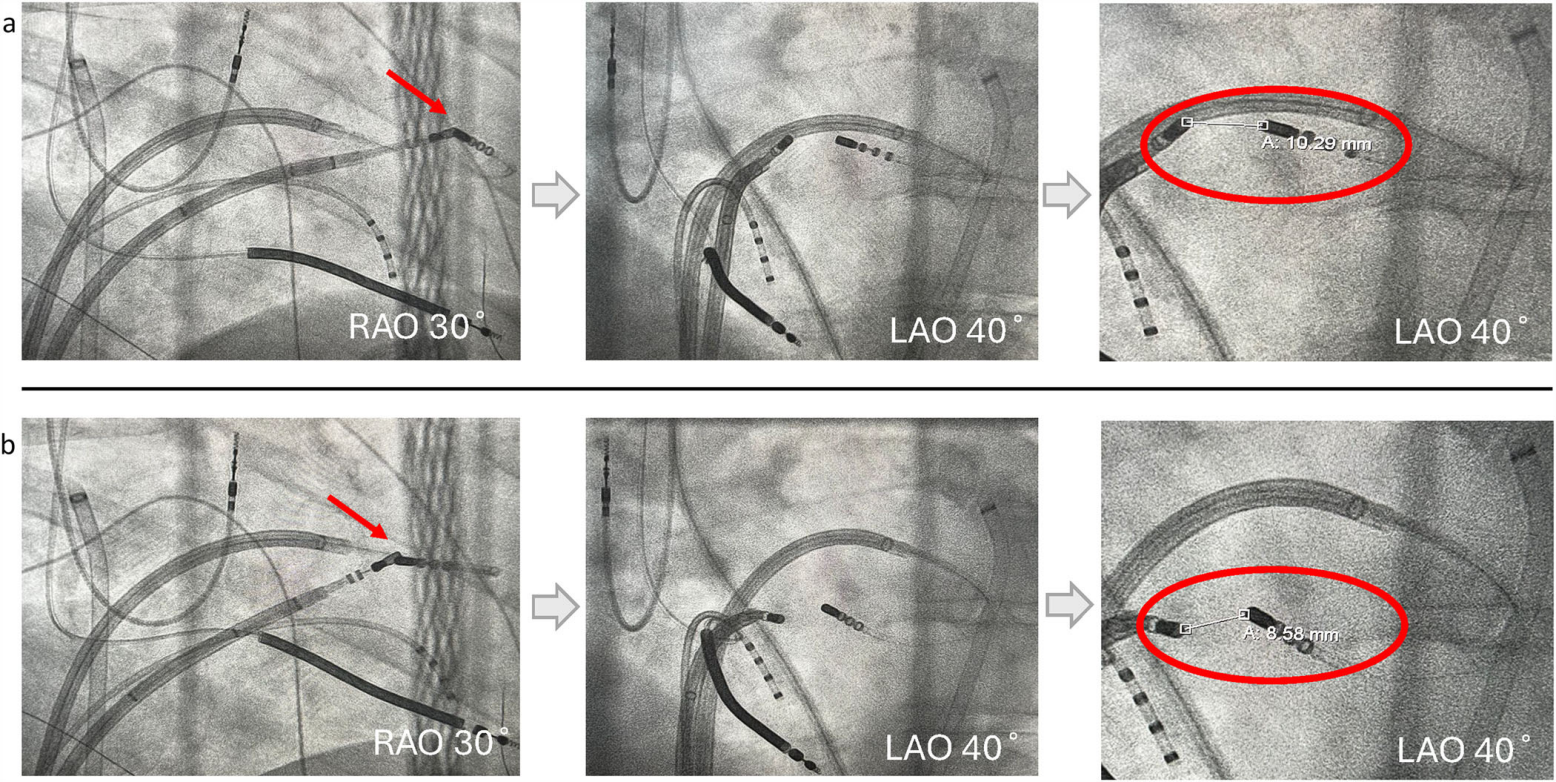
Fluoroscopic images of bipolar ablation at the interventricular septum. Fluoroscopic images of bipolar ablation at the interventricular septum. Two slightly different positions are shown (a and b). Images are used to measure the distance between catheter tips for every bipolar ablation applied.

**Figure 5:**
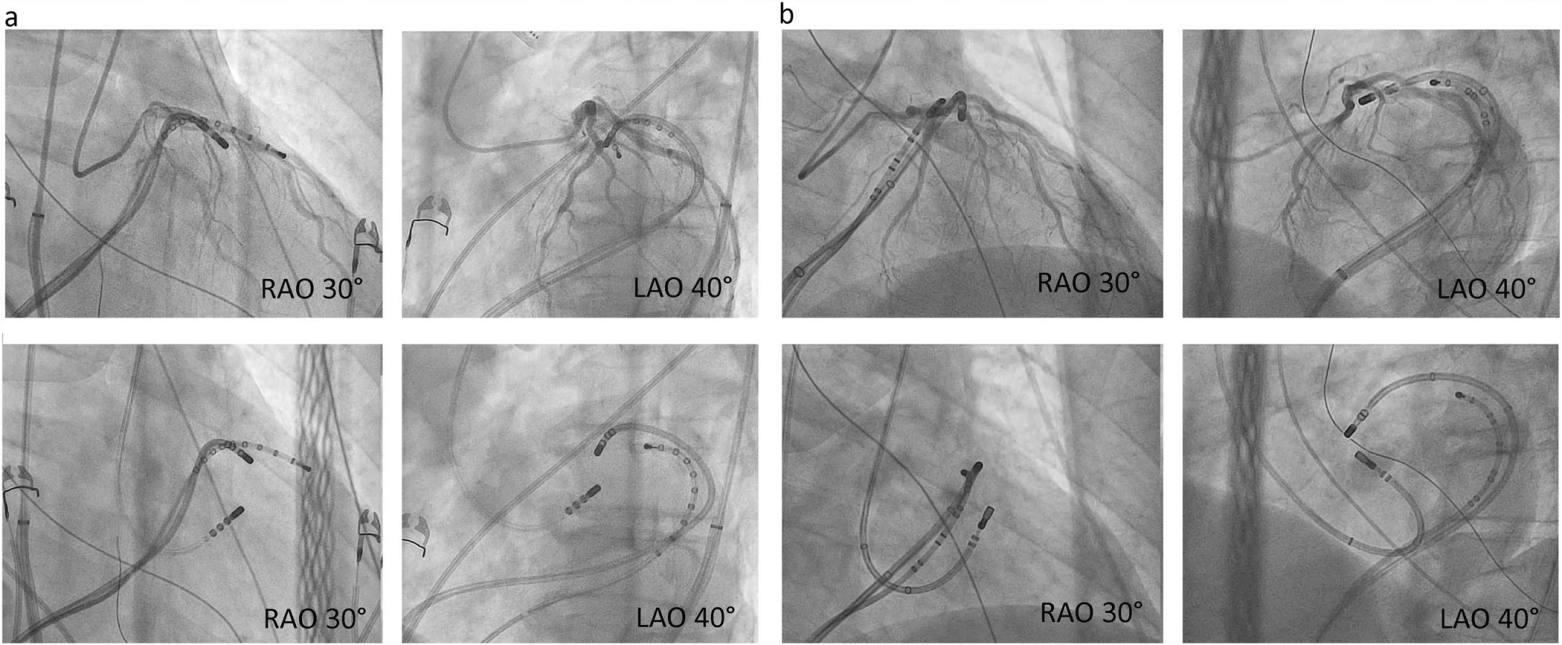
Fluoroscopic images of bipolar ablation at the LV summit. Fluoroscopic images of bipolar ablation at the LV summit in two procedures (a and b) with the coronary angiogram displayed above and the catheter positions for bipolar ablation shown below.

Overall, 226 bipolar ablation lesions were applied, with a mean 11 ± 6 bipolar lesions per patient and a mean duration of 293 ± 206 s. Mean impedance drop was 15% ± 7%. Temperature of the RC was continuously monitored with the use of the second generator. Catheter temperature did not rise above 44°C during bipolar ablation. No overheating of the active or the RC was observed during bipolar catheter ablation. No sudden temperature rise or stream pop was detected. Adequate tissue contact of the AC was ensured by continuous monitoring of the contact force. For the passive catheter, contact force monitoring was not possible, as it was a noncontact force sensing catheter. Stable electrogram morphology and fluoroscopic visualization were used as surrogate parameters to assess adequate tissue contact. Mean distance between the AC and the RC during ablation was 14 ± 5 mm. Distance measurement was performed using fluoroscopic images (Figure 4). In the cases with endo‐epicardial bipolar ablation between distal cardiac vein and the anterobasal LV, the CS catheter was could be positioned directly on the opposite of the endocardial catheter without the need to be moved because of too close proximity to coronary arteries (Figure 5).

**Video 1:** Bipolar septal ablation at VT isthmus site.

Detailed bipolar ablation characteristics are given in Table 3.

**Table 3:** Detailed bipolar ablation characteristics.

Case no.	Procedure	Cardiomyopathy	Target region	Active catheter	Return catheter	No. of bipolar ablations	Bipolar impedance drop	Mean catheter distance (mm)
1	VT	HCM	IVS	Septal RV	Septal LV	8	9 ± 3	12
2	VT	ICM	IVS	Septal LV	Septal LV	6	13 ± 7	11
3	VT storm	ICM	IVS	Septal RV	Septal LV	11	10 ± 4	9
4	PVC	NICM	LV summit	LVOT	CS	7	12 ± 7	11
5	VT storm	NICM	IVS/LV free wall	Epicardial	RV/LV	28	25 ± 13	9
6	VT storm	NICM	IVS	Septal LV	Septal RV	10	7 ± 14	13
7	VT storm	Idiopathic	AL Pap muscle	AL Pap muscle	AL Pap muscle	2	4 ± 0.5	6
8	PVC	Idiopathic	LV summit	CS	LV	8	10 ± 8	6
9	PVC	Idiopathic	LV summit	CS	LV	5	22 ± 11	8
10	PVC	NICM	LV summit	LV	CS	2	16 ± 0.5	7
11	VT	ICM	LV anterior wall	Epicardial, anterior	LV, anterior	14	12 ± 9	14
12	PVC	Idiopathic	LV summit	LV/CS	CS/LV	8	7	6
13	VT	NICM	IVS	Septal LV	Septal RV	15	16 ± 7	13
14	VT	NICM	IVS	Septal LV/RV	Septal RV/LV			
10	8 ± 4	12						
15	VT	ICM	IVS	Septal LV	Septal RV	6	16 ± 3	9
16	VT storm	NICM	IVS	Septal LV/RV	Septal RV/LV	18	16 ± 6	12
17	VT storm	NICM	IVS	Septal LV	Septal RV	16	5 ± 3	13
18	VT storm	ICM	IVS	Septal LV	Septal RV	21	15 ± 8	12
19	VT	ICM	LV, anterior wall	LV, anterior	Epicardial, anterior	6	14 ± 6	12
20	VT	NICM	RV, inferior wall	Epicardial, inferior	Endocardial, inferior	16	11 ± 3	8
21	VT	ICM	IVS	Septal LV	Septal RV	9	24 ± 9	11

Abbreviations: AL, anterolateral; CS, coronary sinus; DCM, dilatative cardiomyopathy; GCV, great cardiac vein; HCM, hypertrophic cardiomyopathy; ICM, ischemic cardiomyopathy; IVS, interventricular septum; LCC, left coronary cusp; LV left ventricle; LVOT left ventricular outflow tract; Pap, papillary; PVC, premature ventricular contraction; RV, right ventricular; VT, ventricular tachycardia.

### 3.4 Acute Procedural Outcome and Complications

Acute success was achieved in 17 of 23 procedures. Mean PVC burden before ablation was 17% with complete elimination in three cases and reduction of PVC burden without complete elimination (= ablation failure) in two cases. In all patients undergoing PVC ablation and suspected intramural substrate, bipolar ablation was carried out initially to avoid superficial edema by initial unipolar ablation. In two patients, presenting with PVCs originating from the LV summit a combined bipolar and unipolar approach failed to completely eliminate PVCs. In the remaining three patients undergoing successful PVC ablation, solely bipolar ablation was performed.

In VT procedures, noninducibility of VT was achieved in 14 of 16 procedures (88%). Bipolar ablation was performed to target the intramural substrate with additional unipolar ablation to address a broader area on the underlying substrate. However, the decision to perform bipolar ablation was not based on failure of initial unipolar ablation, but by suspected intramural substrate, defined by the mapping criteria.

In patients presenting with VT storm and nonischemic cardiomyopathy (ejection fraction 10%), VA reoccurred on the day of procedure despite noninducibility at the end of the procedure. In one patient with preexisting implantable cardioverter‐defibrillator, a permanent atrioventricular block III occurred after bipolar ablation at the high interventricular septum.

No abnormalities, in particular, no ventricular septal defect was detected in any of the patients after the procedure and during follow‐up.

During the follow‐up time of 12 ± 6 months, four patients with initially acute procedural success (24%) experienced recurrence of VT (*n* = 3) or PVC (*n* = 1). Two patients died, one due to septic shock 3 weeks after the procedure and another one due to cardiogenic shock 8 days after the procedure. Both patients, the 81‐year‐old patient with septic shock and the 60‐year‐old patient with nonischemic cardiomyopathy and cardiogenic shock underwent septal bipolar ablation. Both deaths were in‐hospital without discharge after catheter ablation. Both patients underwent repeat transthoracic echocardiography and a ventricular septal defect was ruled out. No association between catheter ablation and the fatal outcome could be detected.

## 4 Discussion

This study presents a large single‐center experience of bipolar catheter ablation in patients undergoing their first ablation procedure or repeat procedure after a previously failed ablation attempt for VAs.

These are the main findings:

1. Bipolar ablation is safe and feasible in patients presenting with intramural arrhythmias.
2. The ablation approach can be performed during the first procedure as well as after a previously failed ablation attempt.
3. Endo‐epicardial bipolar ablation can be a useful tool when intramural arrhythmias are present.

Bipolar ablation was previously reported to be performed as a bail‐out procedure, mainly when the initial ablation procedure failed. Kany and colleagues retrospectively analyzed 26 procedures of 7 different centers for bipolar ablation in patients presenting with VA refractory to conventional therapy, with a median of 1 previous ablation performed before inclusion, with only 2 patients presenting for initial VA ablation [9]. Futyma and colleagues published a large retrospective registry with 93 bipolar ablation procedures of 16 different European centers including only patients after at least one failed standard unipolar ablation attempt [10]. Caixal and colleagues recently reported their single‐center experience of patients undergoing bipolar RF ablation. Of these, 17 (81%) were redo procedures and only 4 patients underwent bipolar RF ablation within their initial procedure [15].

In this current study cohort, 10/21 (48%) of bipolar ablation procedures were performed without any previously failed ablation attempt. The decision to perform bipolar ablation in procedures with and without previously failed ablation therapy was based on different factors identified by electroanatomical mapping. Specific electrocardiogram (ECG) characteristics of PVCs originating from within the interventricular septum with intramyocardial origin have been previously described [16]. These ECG criteria can help identify those patients with PVCs from the interventricular septum, who may benefit from bipolar ablation.

Combined unipolar and bipolar ablation was performed in 86% of cases. In an ex vivo model, it was shown that lesion size significantly increased when combining sequential unipolar and bipolar ablation [9]. However, the potential benefit of performing first unipolar ablation followed by bipolar ablation or vice versa has not been assessed so far. We routinely perform initially bipolar ablation, followed by unipolar ablation. One hypothesis promoting this approach is that initial unipolar ablation may lead to superficial tissue edema, causing less penetration of energy during bipolar catheter ablation.

The correct and early utilization of bipolar ablation may potentially spare the need for a repeat catheter ablation. Another aspect in favor for early bipolar ablation is the safety outcome of the current study cohort. Previously, bipolar ablation was limited by several factors. Specific custom cabling was required and catheter tip temperature could not be monitored simultaneously for both catheters. Catheter–tissue contact was difficult to assess and irrigation was oftentimes used for only one of the two catheters, increasing the risk of coagulation and steam pops.

In the present study cohort, all bipolar ablation procedures were performed using a dedicated bipolar ablation adapter, as previously described [17]. This allowed to connect the passive catheter to a second ablation generator to monitor temperature of both catheter tips throughout ablation. Moreover, irrigation for both catheters could be controlled independently. Using externally irrigated ablation catheters as the active and the ground tips not only produces larger and deeper lesions, as shown by Nguyen et al. [18], but also reduces the risk of adverse events, such as steam pops and subsequent tamponade. The second generator allows for temperature monitoring of the RC. The importance of monitoring temperature values of the intracardiac return electrode was shown by Futyma et al. [12]. No steam pops were observed in this current study population.

Bipolar RFA becomes less effective when the distance between the two catheters becomes too big, by reducing the current density in between both catheters. In this present study, the location of both catheter tips was checked before every bipolar ablation, to assure minimal distance between both catheters. The mean distance for every case did not exceed 20 mm.

Epicardial access and with bipolar ablation was performed in four procedures. Epicardial adipose tissue can limited lesion formation during bipolar ablation [9]. In all cases with epicardial bipolar ablation, both baseline impedance and impedance drop were monitored to assure adequate lesion formation. However, whether this also resulted in similar lesion size cannot be answered. Importantly, the proximity to the coronary arteries and phrenic nerve needs to be assessed prior to ablation.

One pericardio‐esophageal fistula in a patient undergoing bipolar endo‐epicardial RF ablation of the lateral basal LV was reported previously. The patient expired due to massive hematemesis 1 month after procedure. At postmortem, the cause of death was determined to be unrelated to ablation, with evidence of recent bleeding of a gastric ulcer. However, examination of the esophagus revealed a pericardio‐esophageal fistula [19]. In the present study, no bipolar ablation at the lateral basal LV was performed and no pericardio‐esophageal fistula occurred.

In one patient, bipolar catheter ablation led to successful treatment of incessant VT originating from anterolateral papillary muscle. Unipolar ablation at the site of the earliest diastolic potential led to VT termination, however, the VT was still inducible, suggestive of a deep intramural substrate. During bipolar ablation, the catheters were placed on the opposite site and close to the base of the papillary muscle. Contact force sensing of the AC allowed adequate tissue contact, however, no intraprocedural imaging was used for visualization. This case was published earlier as the first report of successful bipolar VT ablation with the involvement of the anterolateral papillary muscle [20]. No peri‐ or postprocedural complications occurred and during the 1‐year follow‐up, the patient was free from VT.

## 5 Limitation

This study has several limitations. As we report one of the largest single‐center experiences, the total number of procedures remains small. Moreover, no control group was investigated. Furthermore, intracardiac echo was not available in any of the cases. The use of intracardiac echo can be beneficial for achieving more stable contact especially in myocardial structures, such as the papillary muscles. Contact force sensing catheters were used in all cases to assure stable contact‐tissue contact of the AC. For the passive catheter, contact force monitoring was not possible, as it was a noncontact force sensing catheter. Stable electrogram morphology and fluoroscopic visualization were used as surrogate parameters to assess adequate tissue contact.

In only a small number of cases, endo‐epicardial bipolar ablation of the LV or right ventricular free wall was performed. More data is needed to asses, whether bipolar approach is a useful tool when intramural arrhythmias are present in these areas.

## 6 Conclusion

Bipolar catheter ablation alone or in combination with unipolar ablation can be an effective tool by targeting different arrhythmias with an intramural origin. It is shown to be safe with a high acute success rate in patients undergoing their first or redo procedure for VT or PVC.

## Conflicts of Interest

The authors declare no conflicts of interest.

## Data Availability

The data that support the findings of this study are available from the corresponding author upon reasonable request.
